# Factors associated with stress, anxiety, and depression during social distancing in Brazil

**DOI:** 10.11606/s1518-8787.2021055003152

**Published:** 2021-03-31

**Authors:** Alex Sandro Rolland Souza, Gustavo Fonseca Albuquerque Souza, Gabriela Albuquerque Souza, Ana Lorena Nascimento Cordeiro, Gabriella Almeida Figueredo Praciano, Adricia Cristine de Souza Alves, Alan Chaves dos Santos, José Roberto Silva, Manuela Barbosa Rodrigues Souza

**Affiliations:** I Instituto de Medicina Integral Prof. Fernando Figueira Centro de Atenção à Mulher RecifePernambuco Brasil Instituto de Medicina Integral Prof. Fernando Figueira. Centro de Atenção à Mulher. Recife, Pernambuco, PE, Brasil; II Universidade Federal de Pernambuco Departamento Materno Infantil RecifePernambuco Brasil Universidade Federal de Pernambuco. Departamento Materno Infantil. Recife, Pernambuco, PE, Brasil; III Universidade Católica de Pernambuco Centro de Ciências Biológicas e da Saúde RecifePernambuco Brasil Universidade Católica de Pernambuco. Centro de Ciências Biológicas e da Saúde. Recife, Pernambuco, PE, Brasil; IV Faculdade Pernambucana de Saúde RecifePernambuco Brasil Faculdade Pernambucana de Saúde. Recife. Pernambuco, PE, Brasil

**Keywords:** Mental Disorders, epidemiology, Stress, Psychological, Social Isolation, Coronavirus Infections, Health Surveys

## Abstract

**OBJECTIVE:**

To estimate the prevalence of clinical signs and symptoms of severe/extreme stress, anxiety, and depression, as well as their associated factors, among Brazilians during social distancing.

**METHODS:**

This is a cross-sectional study conducted in April/May 2020 with 3,200 Brazilians over 18 years old. Respondents’ sociodemographic and clinical data were collected using an online questionnaire, which also included the 21-item Depression, Anxiety and Stress Scale (DASS-21) to assess emotional symptoms. Unadjusted and adjusted prevalence ratios and their respective 95% confidence intervals were estimated using Poisson regression models with robust variance.

**RESULTS:**

Our results show the prevalence of severe/extreme stress was 21.5%, anxiety 19.4%, and depression 21.5%. In the final model, sociodemographic, clinical, and Covid-19-related factors were associated with severe/extreme stress, anxiety, and depression in Brazilians during social distancing due to the Covid-19 pandemic. We found the main factors associated with severe/extreme depression to be young women, brown, single, not religious, sedentary, presenting reduced leisure activities, history of anxiety and depression, increased medication use, and Covid-19 symptoms.

**CONCLUSION:**

This study may help develop and systematically plan measures aimed to prevent, early identify, and properly manage clinical signs and symptoms of stress, anxiety, and depression during the Covid-19 pandemic.

## INTRODUCTION

Emerging infectious disease in humans may require preventive measures to contain infections spread. However, these measures tend to influence individuals’ behavior and interaction with each other^1^. Among the recommendations to prevent severe acute respiratory syndrome coronavirus-2 (SARS-CoV-2) spread^1^, social distancing has proved to be effective in containing the disease^2^.

Studies have shown that social distancing affects mental health, constituting a risk factor for mental disorders^1^. Mental well-being plays a seminal role in the overall health, so that mental disorders incur functional disabilities, reducing individuals’ quality of life, increasing healthcare-related costs, and damaging interpersonal relationships^3^.

Recent evidence suggests the Covid-19 pandemic has a psychological impact on individuals^4^, be it direct or indirect. Directly, the pandemic affects individuals’ mental health through the virus neurotropic properties, and indirectly through excessive concern over one’s future during social distancing^5^. However, studies in different countries still need to be consolidated for providing more information on the subject.

Before the pandemic, the prevalence of anxiety disorders was 9.3% in Brazil, whereas for depression this index was 5.4%, ranging from mild to extreme^6^. However, the literature suggests that Covid-19 crisis increased these numbers, as the country recorded a higher prevalence of depression (61.3%), anxiety (44.2%), stress (50.8%), and psychological impact (54.9%) during the pandemic^7^. Likewise, China, the first country to adopt social distancing measures against the virus spread, reported rates of psychological distress ranging from 7% to 53.8% during the initial stage of the Covid-19^4^.

According to studies conducted during social distancing, the main risk factors for stress, anxiety, and depression are being female, a healthcare worker, over 50 years old, having poor education level, presenting some type of physical morbidity or psychiatric disorder, and having a friend or relative with Covid-19 symptoms^8^.

Considering the importance of data availability in preventing mental disorders, the exponential increase in the number of cases and deaths in Brazil, and the implemented social distancing measures, this study evaluated the prevalence of severe/extreme stress, anxiety, and depression in the Brazilian population and determined the factors associated with these symptoms during the Covid-19 pandemic. Added to other studies on the same theme^7,11,12^, our results should be considered as a whole for planning and developing health actions aimed at this population. Moreover, we explored risk factors not previously addressed in the literature, be it national^7,11,12^ or international^4,8^.

## METHODS

This is a prospective cross-sectional study conducted in April and May 2020. Native or naturalized Brazilians living in Brazil or abroad were eligible for inclusion. Individuals who failed completing the questionnaire and those under 18 years old were excluded.

To collect participants’ sociodemographic and clinical data, an online questionnaire with easily understandable objective-response questions was developed using the Google Forms platform, including the 21-item Depression, Anxiety and Stress Scale (DASS-21) validated for Brazil^13^.First, all investigators involved in the study sent the questionnaire to their contacts via Instagram, Facebook, WhatsApp, and Twitter, and then asked those to forward the survey to all their contacts on social media, advertising the research.

Independent variables consisted of participants’ sociodemographic data, such as age, gender, race/ethnicity, education level, marital status, geographic region of residence, individuals per household, number of children, rooms per household, and religion.

Participants also reported their activities – including the practice of leisure activities, physical exercise, and Internet usage before and during social distancing – and whether they have a medical history of anxiety and depression, chronic diseases, illicit drug use, smoking, alcohol consumption, and medication use. Regarding work-related activities, participants were asked about their occupation, employment status, monthly income before and after social distancing measures implementation, and whether they were able to work from home.

Based on a previous research, this study nominally categorized the variables practice leisure activities, physical exercise, internet usage, smoking, alcohol consumption, medication use, and working from home during social distancing as “increased,” “reduced,” “I do not perform/use,” or “remains the same”^14^.

Respondents also reported the preventive measures adopted by them aimed at preventing SARS-CoV-2, such as quarantine, social distancing, or social isolation^1^, as well as social distancing duration. The questionnaire also contained disease-related questions, such as whether any family member or acquaintance have been infected with the disease, whether they had contact with someone suspected or confirmed to have it, and whether themselves presented symptoms, as well as any test results on the disease.

Dependent variables consisted of clinical signs and symptoms of stress, anxiety, and depression, according to each subscale of DASS-21, used to identify emotional symptoms. Based on a 4-point Likert-type scale, DASS-21 required participants to report the degree to which they had experienced each symptom (described in statements) over the preceding week. In the stress subscale, scores ranging from 0–14 indicate normal, from 15–18 mild symptoms, from 19–25 moderate, from 26–33 severe symptoms, and > 33 indicate extremely severe symptoms. As for the anxiety subscale, scores ranging from 0–7 are classified as indicative of normal, 8–9 mild, 10–14 moderate symptoms, 15–19 severe, and > 19 extremely severe symptoms. Finally, in the depression subscale, scores ranging from 0–9 are indicative of normal results, 10–13 mild symptoms, 14–20 moderate, 21–27 severe, and > 27 extremely severe symptoms^13^.

Sample size was determined using the Epi Info StatCalc software version 3.5.1 (Centers for Disease Control and Prevention – CDC, United States of America – USA, Atlanta, DC). Predicting a 5% rate of depression among Brazilians^9^ (WHO, 2017) and considering a 80% power and a 95% significance level, 810 patients will be necessary for the study.

All analyses were performed on Stata version 12.1 (StataCorp, College Station, USA). First, a univariable analysis was conducted to describe the dependent and independent variables distribution in the study population. Unadjusted and adjusted prevalence ratio (PR) and their respective 95% confidence intervals (95%CI) were calculated using Poisson regression with robust variance. The significance of each explanatory variable present in the model was evaluated using the Wald test. In the multivariable analysis, the initial model consisted of the explanatory variables with p < 0.20 in the univariable analysis; variables with significance level < 0.05 remained in the final model.

This study was approved by the institutional review board of the Universidade Católica de Pernambuco, under reference number 3.988.875 on April 24, 2020 and CAAE 30623020.1.0000.5206. All participants received an online informed consent form and checked either “sign” or “do not sign,” so that only those who agreed to participate and chose to sign were included in the study.

## RESULTS

Of the 3,793 individuals who responded the questionnaire, 3,200 were included in the study (84.4%). We excluded the forms of 565 respondents (14.9%) who failed in fully completing the questionnaire or who answered it in duplicate, and of 28 (0.7%) who were underage.

Regarding participants’ epidemiological characteristics, we verified that 21.3% (n = 681) were aged between 34 and 44 years, 45.2% (n = 1446) were catholic, and 7.0% (n = 223) reported to have been socially distancing for 10 days or less. Most respondents were women (n = 2473; 77.3%), white (n = 1929; 60,3%), single (n = 1688; 52,8%), undergraduate students (n = 1759; 55,0%), and lived in the North-Eastern region of Brazil (n = 2411; 75,3%) (Table 1).

**Table 1:** Participants’ biological and sociodemographic data and social distancing duration (n = 3,200).

	n	%
Age (years)		
18–20	569	17.8
21–25	666	20.8
26–33	627	19.6
34–44	681	21.3
45–83	657	20.5
Sex		
Male	727	22.7
Female	2,473	77.3
Skin color/ethnicity		
White	1,929	60.3
Brown	1,015	31.7
Black	198	6.2
Other	58	1.8
Education level		
Postgraduate	1,105	34.5
Undergraduate student	1,759	55.0
Primary/secondary school	336	10.5
Marital status		
Married	1,253	39.2
Single	1,688	52.8
Others	259	8.0
Region of the country in which respondent lives		
Northeast	2,411	75.3
Southeast	430	13.4
South	158	4.9
North	96	3.0
Midwest	75	2.3
Abroad	30	0.9
Religion		
Catholic	1,446	45.2
Evangelical	497	15.5
Not religious	793	24.8
Other	464	14.5
Duration of social distancing (days)		
≤ 10	223	7.0
11–30	815	25.5
31–35	290	9.1
36–40	1,022	31.9
41–45	538	16.8
46–122	312	9.7

Based on symptoms severity, we found the prevalence rate of stress within the study population to be equal to 21.5%, of anxiety to be qual to 19.4%, and depression to 21.5% (Table 2).

**Table 2:** Prevalence rates of stress, anxiety, and depression according to severity (n = 3,200).

	Normal		Mild		Moderate		Severe		Extreme	
	n	%	n	%	n	%	n	%	n	%
Stress	1,679	52.5	414	12.9	419	13.1	413	12.9	275	8.6
Anxiety	1,774	55.4	252	7.9	552	17.3	203	6.3	419	13.1
Depression	1,496	46.8	414	12.9	602	18.8	252	7.9	436	13.6

In the final model, risk factors associated with clinical signs and symptoms of severe/extreme stress were: age between 18 and 20 years (PR: 4.49; 95%CI: 3.31–6.09; p < 0.001); being of female gender (PR: 1.67; 95%CI: 1.37–2.02; p < 0.001); living abroad (PR: 2.03; 95%CI: 1.31–3.16; p = 0.002); having a history of anxiety and depression (PR: 2.14; 95%CI: 1.84–2.49; p < 0.001); having reduced leisure activity practice (PR: 1.71; 95%CI: 1.44–2.02; p < 0.001); having increased medication use (PR: 1.97; 95%CI: 1.70–2.27; p < 0.001); and complying with social isolating measures (PR: 1.36; 95%CI: 1.14–1.63; p < 0.001). Conversely, being able to work from home (PR: 0.77; 95%CI: 0.63–0.94; p = 0.011) and using the Internet for leisure activities (PR: 0.78; 95%CI: 0.65–0.93; p = 0.005) were protective factors against stress (Table 3 and Table 3S).

**Table 3:** Factors associated with severe/extreme stress during social distancing in Brazil (n = 3,200).

Factors	Final Model	
	PR (95%CI)	p*
Age (years)		< 0.001
18–20	4.49 (3.31–6.09)	< 0.001
21–25	3.97 (2.93–5.39)	< 0.001
26–33	2.91 (2.13–3.97)	< 0.001
34–44	1.84 (1.32–2.57)	< 0.001
45–83	1.0 (Ref.)	
Sex		< 0.001
Male	1.0 (Ref.)	
Female	1.67 (1.37–2.02)	< 0.001
Working from home		0.006
Increased	1.0 (Ref.)	
Remains the same	0.77 (0.63–0.94)	0.011
Decreased	1.14 (0.98–1.32)	0.090
I do not work from home	1.09 (0.90–1.31)	0.380
Region of the country in which respondent lives		< 0.001
Northeast	1.0 (Ref.)	
Southeast	1.52 (1.30–1.78)	< 0.001
South	1.27 (1.03–1.56)	0.025
North	1.28 (0.93–1.77)	0.123
Midwest	1.54 (1.10–2.16)	0.012
Abroad	2.03 (1.31–3.16)	0.002
History of anxiety and depression		< 0.001
No	1.0 (Ref.)	
Yes	2.14 (1.84–2.49)	< 0.001
Leisure activities		< 0.001
Increased	1.0 (Ref.)	
Reduced	1.71 (1.44–2.02)	< 0.001
Remain the same	1.00 (0.84–1.18)	0.966
I do not perform leisure activities	1.33 (0.83–2.14)	0.241
Internet use		0.005
Increased	1.0 (Ref.)	
Remains the same	0.78 (0.65–0.93)	0.005
Reduced/I do not use the Internet	1.23 (0.93–1.63)	0.147
Use of medication		< 0.001
I do not use any medication	1.0 (Ref.)	
Use increased	1.97 (1.70–2.27)	< 0.001
Remains the same	1.10 (0.90–1.35)	0.333
Use decreased	0.84 (0.46–1.54)	0.577
Social isolation		< 0.001
No	1.0 (Ref.)	
Yes	1.36 (1.14–1.63)	< 0.001

* Poisson regression.

Regarding severe/extreme anxiety, risk factors were: being of female gender (PR: 1.90; 95%CI: 1.54–2.34; p < 0.001); being brown-skinned (PR: 1.60; 95%CI: 1.31–1.94; p < 0.001); having reduced possibility of working from home (PR: 1.37; 95%CI: 1.17–1.60; p < 0.001); living in the North region of the country (PR: 1.42; 95%CI: 1.07-1.90; p = 0.017); having no more than 1–6 rooms within the household (PR: 1.23; 95%CI: 1.08–1.40; p = 0.002); having a history of anxiety and depression (PR: 2.57; 95%CI: 2.18–3.04; p < 0.001); having a chronic disease (PR: 1.24; 95%CI: 1.08–1.41; p = 0.002); having reduced leisure activity practice (PR: 1.44; 95%CI: 1.20–1.74; p < 0.001); having increased medication use (PR: 1.96; 95%CI: 1.68–2.29; p < 0.001); and complying with social isolating measures (PR: 1.26; 95%CI: 1.04–1.52; p = 0.019) or quarantine (PR: 1.19; 95%CI: 1.02–1.40; p = 0.031). Protection against severe/extreme anxiety increased alongside age, specially within the 45-83-year age group (PR: 0.27; 95%CI: 0.19–0.36; p < 0.001) (Table 4 and Table 4S).

**Table 4:** Factors associated with severe/extreme anxiety during social distancing in Brazil (n = 3,200).

Factors	Final model	
	PR (95%CI)	p*
Age (years		< 0.001
18–20	1.0 (Ref.)	
21–25	0.89 (0.75–1.05)	0.169
26–33	0.76 (0.63–0.91)	0.003
34–44	0.40 (0.32–0.50)	< 0.001
45–83	0.27 (0.19–0.36)	< 0.001
Sex		< 0.001
Male	1.0 (Ref.)	
Female	1.90 (1.54–2.34)	< 0.001
Skin color/ethnicity		< 0.001
White	1.0 (Ref.)	
Brown	1.60 (1.31–1.94)	< 0.001
Black	0.80 (0.38–1.66)	0.543
Other	1.19 (1.03–1.38)	0.016
Working from home		< 0.001
Increased	1.0 (Ref.)	
Remains the same	0.90 (0.73–1.10)	0.306
Decreased	1.37 (1.17–1.60)	< 0.001
I do not work from home	1.28 (1.05–1.56)	0.014
Region of the country in which respondent lives		0.018
Northeast	1.0 (Ref.)	
Southeast	1.29 (1.08–1.54)	0.004
South	1.14 (0.88–1.46)	0.317
North	1.42 (1.07–1.90)	0.017
Midwest	1.14 (0.82–1.59)	0.421
Abroad	1.51 (0.90–2.53)	0.123
Number of rooms in the house		0.002
1–6	1.23 (1.08–1.40)	0.002
7 or more	1.0 (Ref.)	
Religion		0.010
Catholic	1.0 (Ref.)	
Evangelical	0.85 (0.69–1.04)	0.107
Not religious	1.12 (0.96–1.30)	0.158
Other	0.82 (0.66–1.01)	0.063
History of anxiety and depression		< 0.001
No	1.0 (Ref.)	
Yes	2.57 (2.18–3.04)	< 0.001
Chronic diseases		0.002
No	1.0 (Ref.)	
Yes	1.24 (1.08–1.41)	0.002
Leisure activities		0.002
Increased	1.0 (Ref.)	
Reduced	1.44 (1.20–1.74)	< 0.001
Remain the same	1.02 (0.86–1.22)	0.783
I do not perform leisure activities	1.18 (0.74–1.88)	0.478
Use of medication		< 0.001
I do not use any medication	1.0 (Ref.)	
Use increased	1.96 (1.68–2.29)	< 0.001
Remains the same	1.11 (0.90–1.38)	0.324
Use decreased	0.88 (0.48–1.60)	0.670
Quarantine		0.031
No	1.0 (Ref.)	
Yes	1.19 (1.02–1.40)	0.031
Social isolation		0.019
No	1.0 (Ref.)	
Yes	1.26 (1.04–1.52)	0.019

* Poisson regression.

As for clinical signs and symptoms of severe/extreme depression, risk factors were: being aged between 21 and 25 years (PR: 2.77; 95%CI: 1.93–3.97; p < 0.001); being of female gender (PR: 1.44; 95%CI: 1.21–1.71; p < 0.001); being brown-skinned (PR: 1.40; 95%CI: 1.13–1.73; p = 0.002); being single (PR: 1.52; 95%CI: 1.27–1.83; p < 0.001); presenting decreased family income (PR: 1.20; 95%CI:1.06–1.36; p = 0.004); having reduced possibility of working from home (PR: 1.50; 95%CI: 1.29–1.75; p < 0.001); being unemployed (PR: 1.60; 95%CI: 1.20–2.13; p = 0.001); living abroad (PR: 2.48; 95%CI: 1.66–3.69; p < 0.001); having no more than 1–6 rooms within the household (PR: 1.21; 95%CI: 1.07–1.37; p = 0.002); not being religious (PR: 1.33; 95%CI: 1.14–1.54; p < 0.001); having a history of anxiety and depression (PR: 1.68; 95%CI: 1.45–1.93; p < 0.001); having reduced leisure activity practice (PR: 1.45; 95%CI: 1.20–1.74; p < 0.001); having reduced physical activity practice (PR: 1.27; 95%CI: 1.06–1.52; p = 0.010); reduced or null Internet usage (PR: 1.36; 95%CI: 1.03–1.78; p < 0.029); having increased medication use (PR: 2.25; 95%CI: 1.94–2.59; p < 0.001); using illicit drug (PR: 1.26; 95%CI: 1.06-1.49; p = 0.008); complying with social isolation measures (PR: 1.21, 95%CI: 0.99-1.49; p < 0.059); and presenting Covid-19 symptoms (PR: 1.37; 95%CI: 1.12-1.68; p = 0.002) (Table 5 and Table 5S).

**Table 5:** Factors associated with severe/extreme depression during social distancing in Brazil (n = 3,200).

Factors	Final Model	
	PR (95%CI)	p*
Age (years)		< 0.001
18–20	2.67 (1.85–3.84)	< 0.001
21–25	2.77 (1.93–3.97)	< 0.001
26–33	2.50 (1.78–3.50)	< 0.001
34–44	1.66 (1.17–2.36)	0.004
45–83	1.0 (Ref.)	
Sex		< 0.001
Male	1.0 (Ref.)	
Female	1.44 (1.21–1.71)	< 0.001
Skin color/ethnicity		0.014
White	1.0 (Ref.)	
Brown	1.40 (1.13–1.73)	0.002
Black	1.25 (0.76–2.07)	0.383
Other	1.00 (0.87–1.15)	0.980
Marital status		< 0.001
Married	1.0 (Ref.)	
Single	1.52 (1.27–1.83)	< 0.001
Other	1.42 (1.07–1.89)	0.016
Family income		0.004
Has not decreased	1.0 (Ref.)	
Decreased	1.20 (1.06–1.36)	0.004
Working from home		< 0.001
Increased	1.0 (Ref.)	
Remains the same	1.19 (0.99–1.42)	0.068
Decreased	1.50 (1.29–1.75)	< 0.001
I do not work from home	1.34 (1.10–1.63)	0.003
Employment status		0.006
Employed, working on-site	1.0 (Ref.)	
Employed, working from home	1.28 (0.96–1.70)	0.091
Employed, furloughed	1.54 (1.09–2.17)	0.013
Student, remote learning	1.57 (1.18–2.11)	0.002
Student, no remote learning	1.26 (0.93–1.71)	0.140
Unemployed	1.60 (1.20–2.13)	0.001
Other	1.50 (1.09–2.05)	0.013
Region of the country in which respondent lives		< 0.001
Northeast	1.0 (Ref.)	
Southeast	1.68 (1.44–1.96)	< 0.001
South	1.48 (1.18–1.86)	< 0.001
North	1.13 (0.82–1.55)	0.465
Midwest	1.95 (1.50–2.55)	< 0.001
Abroad	2.48 (1.66–3.69)	< 0.001
Number of rooms in the house		0.002
1–6	1.21 (1.07–1.37)	0.002
7 or more	1.0 (Ref.)	
Religion		< 0.001
Catholic	1.0 (Ref.)	
Evangelical	0.95 (0.78–1.16)	0.609
Not religious	1.33 (1.14–1.54)	< 0.001
Other	1.00 (0.82–1.21)	0.984
History of anxiety and depression		< 0.001
No	1.0 (Ref.)	
Yes	1.68 (1.45–1.93)	< 0.001
Leisure activities		0.001
Increased	1.0 (Ref.)	
Reduced	1.45 (1.20–1.74)	< 0.001
Remain the same	1.02 (0.86–1.21)	0.807
I do not perform leisure activities	1.14 (0.72–1.82)	0.574
Physical exercise		0.002
Increased	1.0 (Ref.)	
Reduced	1.27 (1.06–1.52)	0.010
Remains the same	0.94 (0.74–1.19)	0.605
I do not practice physical exercise	1.29 (1.07–1.57)	0.008
Internet Use		0.025
Increased	1.0 (Ref.)	
Remains the same	1.36 (1.03–1.78)	0.029
Reduced/I do not use the Internet	0.89 (0.76–1.05)	0.163
Use of medication		< 0.001
I do not use any medication	1.0 (Ref.)	
Use increased	2.25 (1.94–2.59)	< 0.001
Remains the same	1.42 (1.17–1.73)	< 0.001
Use decreased	1.56 (0.99–2.48)	0.058
Illicit drug use		0.008
No	1.0 (Ref.)	
Yes	1.26 (1.06–1.49)	0.008
Social isolation		0.059
No	1.0 (Ref.)	
Yes	1.21 (0.99–1.49)	0.059
Symptoms of Covid-19		0.002
Yes	1.37 (1.12–1.68)	0.002
No	1.0 (Ref.)	

* Poisson regression.

## DISCUSSION

We found high prevalence rates of clinical signs and symptoms of severe/extreme stress, anxiety, and depression during the social distancing period recommended to prevent SARS-CoV-2 spread. Moreover, several factors were common to all three mental syndromes.

Four different pillars may explain this finding: the virus, information, sociability, and susceptibility. Studies suggest that psychological stress increases alongside infection and fatality rates. Besides that, while the access to accurate information is limited, fake news are readily available^17,18^. Investigators believe that traumatic events resulting from social distancing reduce the sense of safety, reminding people of death and negatively affecting mental health, given that many questions related to the pandemic are still unanswered^19^. Triggers such as social distancing are more easily pulled among individuals more susceptible to mental disorders, leading to more frequent crises^1^. Therefore, Brazilian researchers suggest that social distancing is not a risk factor for psychological symptoms, and that both social context and the four pillars aforementioned must be analyzed^11^.

The prevalence rates of signs and symptoms of severe/extreme stress, anxiety, and depression were 21.5%, 19.4% and 21.5%, respectively. These rates corroborate a Spanish study that reported prevalence rates of 18.7% for depression and 21.6% for anxiety, without classifying the disorders according to severity^20^. However, our results contradict those reported by a Chinese study, with rates of 8.1% for moderate to severe stress, 28.8% for anxiety, and 16.5% for depression^8^, although different prevalence rates were recorded in the same country^7,11,12^. Brazil registered a high prevalence of depression (61.3%), anxiety (44.2%), stress (50.8%), and psychological impact (54.9%)^7^, similar to those found in this study when combining mild, moderate, and severe cases. These differences may stem from varying contexts between countries, distinct regions within the same country, or from different methodologies and approaches to measure clinical signs and symptoms. Our research was conducted during a period of exponential increase in the number of cases and deaths in Brazil (ranking 4th worldwide regarding the number of cases), with more than 240 thousand cases and 16 thousand deaths^21^.

Studies suggest that older individuals have greater risk of morbidity and mortality related to Covid-19, besides being more prone to mental disorders (according to a study that reported an elevated risk among individuals older than 50 years)^6,22^. Our study, on the other hand, verified a greater prevalence of mental disorders among younger individuals (under 45 years old) and undergraduate students (both in general or considering those able to participate in remote learning). We also found older age to function as a protective factor against mental disorders. A study conducted in a similar setting also reported a greater psychological impact on younger individuals^20,23^, which may be explained by the lower maturity level among this population and their fewer resources to deal with social distancing.

We found women to be more likely to suffer from severe/extreme stress, anxiety, and depression. This finding is corroborated by studies conducted in Brazil^7,11,12^ and other countries, which report a greater risk among women in similar social distancing conditions^22,24^. This might be explained by women’s socially imposed role as family caregivers, making them assume household chores besides their occupation, which overburden them physically and psychologically and increase their susceptibility to mental disorders^9,19,20^. A study conducted prior to the pandemic already demonstrated that women were more likely to develop psychological disorders, either as a result of their double workload and/or hormonal influences^16^_._

Although individuals’ skin color/ethnicity tend to affect their educational, economic, and social opportunities, no consensus has been reached regarding mental disorders association with different ethnic groups and labels. We found self-reported brown skin to constitute a risk factor for severe/extreme anxiety and depression. A systematic review conducted in Brazil, in which race was dichotomized into “white” or “non-white,” found “non-white” individuals to be associated with mental disorders^25^. According to the authors, this may be explained by a history of discrimination and its enduring consequences, which are social factors that increase the risk of psychological symptoms among those “non-white”^25^.

Besides the social distancing measures imposed by Covid-19 pandemic, undergraduate students also had to face the suspension of face-to-face classes and the adoption of remote learning, preventive measures implemented in higher education institutions. This context made these students live a period of uncertainty regarding the future of their education^23^ – an important factor, given that this group already presents a high prevalence rate for psychological disorders in ordinary situations^26,27^.

Recent studies showed that married individuals have better health conditions than widowed, divorced, and single people^28^. Our results indicate a statistically significant association between loneliness and clinical signs and symptoms of stress, anxiety, and depression. A different study found sense of belonging, well-being, and acts of self-compassion to function as protective factors against these disorders^20^. A study conducted prior to social distancing found family stability (which may include marital status and financial stability) to cause positive effects on mental health^22^. Corroborating these findings, we verified that being single or widowed and unemployed incur a higher risk of severe/extreme psychological symptoms.

Although studies conducted outside the social distancing context reported conflicting results regarding financial aspects^29,30^, we found reduction in family income during the pandemic to pose a risk factor for severe/extreme depression. Considering that all social classes may experience reduction in family income, this finding reflects economic instability rather than low-socioeconomic status^10,31^.

As for employment status, individuals who were employed prior to social distancing and were furloughed (although still employed) were considered to be at a risk of severe/extreme depression. Given that most of our study sample worked in the healthcare sector, we expected those who continued working to be in a risk group. A previous study found healthcare professionals to present a high risk for psychiatric disorders during this period due to increased feelings of vulnerability or loss, concerns with patients’ and family members’ health, virus spread, and changes in work and family routines^29^.

Individuals living in the Southeast region of Brazil were at greater risk of developing mental disorders than those living in the Northeastern. Corroborating our findings, a study conducted in California showed that continuous exposure to Covid-19-related information, especially in the major urban centers, increases stress and anxiety rates^18^. This may also be explained by the fact that living at the epicenter of the disease could entail a risk factor for mental disorders. The current epicenter in Brazil is the city of São Paulo, located in the southeastern region. Living abroad was yet another relevant risk factor for stress and severe/extreme depression among Brazilians, increasing alongside the period of separation, possibly as a consequence of being distant from family.

Religiosity plays a pivotal psychological role in believers’ lives by giving meaning to physical and psychological stress and providing comfort and support, particularly in difficult times^32^. We found people who reported following no religion to be at a greater risk of presenting clinical signs and symptoms of severe/extreme depression. These results are supported by the literature, which indicates that religious involvement is correlated with better recovery and improve the ability to manage physical and mental illness^33,34^, especially during crises such as the Covid-19 pandemic. In this study, religion was self- stated, regardless of individuals’ degree of involvement.

We found individuals with previous history of anxiety and depression to present a greater risk of developing or worsening psychological symptoms during the pandemic. This finding is corroborated by another study, which suggests that this context is marked by boredom, disappointment, and irritability^35^. These feelings trigger self-medication or excessive medication use, risk factors for the three mental disorders addressed in the study due to the increased vulnerability caused by the indiscriminate use of benzodiazepines^36^.

The presence of chronic diseases was significantly associated with clinical signs and symptoms of severe/extreme anxiety among our study sample, corroborating the results of a cross-sectional study conducted in China at the beginning of the year^23^. This might be explained by the fact that underlying diseases are strongly associated with severe forms of Covid-19 and increased hospital mortality.

The practice of physical and leisure activities should be strongly encouraged during the pandemic context. We found participants who did not perform leisure or physical activities, as well as those who reduced these practices, to present a greater risk of mental disorders. The literature suggests that physical exercise and leisure activities appear to be interconnected, establishing an association between individuals’ cognitive and physical functioning and thus improving mental health^3^. These findings are corroborated by a meta-analysis on the effects of exercise on mental disorders, which reported that physical activity could effectively control depression due to the excitatory effect on monoamine neurotransmitters and endorphins, and possibly improve self-esteem, self-perception, and personal satisfaction^37^.

As individuals in social isolation or quarantine are precisely those contaminated with or exposed to Covid-19, confinement necessarily results in separation, particularly from family members, loss of personal freedom, and changes in life habits^1^, added to the distress, fear, and implications of the disease, as well as frustration toward helplessness, boredom, and sadness^1^.

Studies found individuals who have or have had Covid-19 symptoms and those who had a family member or close relative diagnosed with the disease and shared the experience with them to be positively associated with anxiety, depression, and post-traumatic stress disorder (PTSD)^20,29^. In our study, individuals presenting symptoms, even without a confirmed diagnosis, were at greater risk of severe/extreme depression, suggesting that this new, unknown infection arise negative emotions, such as fear and anxiety.

Our study questionnaire comprised two facets: the 21-item Depression, Anxiety and Stress Scale (DASS-21) validated for use in the Brazilian population; and other questionnaires collecting sociodemographic data, lifestyle habits, social distancing, and Covid-19. This second set of questionnaires was created by the authors based on a bibliographic research conducted in updated databases from regional, national, and international health agencies^7,13^. However, our study has some limitations inherent to online data collection. The online approach of data collection was the most viable option in the current scenario, for preventing infection spread across investigators and the study population. A strength provided by such approach is that responses were obtained from all regions of the country. Although the final sample was heterogenous, respondents from the northeast region (investigators’ place of residence) were more prevalent. Even though 3,200 individuals comprised the study sample, such prevalence suggests the possibility of selection bias, given that the questionnaire was initially disclosed to the researchers’ contacts. However, the limitations imposed by the online data collection are minimized with the strict control over the eligibility criteria and with the sample size obtained.

Yet another limitation to this study was including Brazilians living abroad. Although countries implemented different pandemic policies, all of them had restrictive social measures in place, and Brazilian individuals were facing further risk for being distant from their relatives.

The literature suggests approaches different than that employed by us to questionnaires addressing signs and symptoms of stress, anxiety, and depression, making it difficult for us to compare the results with other studies. On the other hand, studies show these varying approaches have common ground, and the questionnaire was chosen based on researchers’ experience and the country validation.

Considering that our main goal was to determine factors associated to mental disorders during social distancing due to the Covid-19 pandemic, the study sample also included individuals that, despite of the recommendations, might not have been socially distancing, or individuals who complied with the measure for varying length. This occurs either because they were considered essential workers or simply because they did not adhere to the government guidelines. Moreover, the observational study design increases difficulties in controlling for confounding factors. This issue was minimized with a multivariable analysis, which found no significant correlation between social distancing duration and the mental disorders analyzed.

The DASS-21 questionnaire simply evaluates the prevalence of symptoms of stress, anxiety, and depression; it does not function as a diagnostic method. Considering that, a diagnosis on mental disorders requires a specialized medical evaluation.

## CONCLUSION

Our results indicate a high prevalence of clinical signs and symptoms of severe/extreme stress, anxiety, and depression during social distancing, increasing alongside the duration of the confinement period. We found various factors, many of which common to all three mental disorders, to be associated with the social distancing measures imposed during the Covid-19 pandemic.

Being of female gender, living abroad, practicing no leisure and physical activities, and being unemployed were the most relevant factors associated with signs and symptoms of stress, anxiety, and depression. As suggested by Brazilian researchers^38^, variables such as these can be used to develop federally-funded programs aiming at improving mental health by promoting psychotherapy activities and educational and recreational endeavors, stimulating physical exercise, and providing guidelines for the online workspace, particularly for helping women. Authorities should also promote and encourage video calls between Brazilians residing abroad and their friends and family as a means of communicating and establishing emotional bonds.

Our findings point to the need for implementing preventive measures aimed at reducing or stabilizing the prevalence of stress, anxiety, and depression. To avoid aggravating these mental disorders, early and effective action is crucial.
